# Health disparities among indigenous populations in Latin America: a scoping review

**DOI:** 10.1186/s12939-025-02495-2

**Published:** 2025-04-30

**Authors:** Mariana Garza, Lucía Abascal Miguel

**Affiliations:** 1 University of Texas at Austin, Austin, TX USA; 2 University of California, San Francisco, CA USA

**Keywords:** Indigenous populations, Health status disparities, Latin America, Scoping review

## Abstract

**Background:**

Health disparities persist among Indigenous populations in Latin America, reflecting systemic inequities and historical marginalization. These disparities span infectious diseases, malnutrition, and chronic conditions, necessitating a comprehensive understanding to inform equitable public health strategies. This scoping review aims to map health disparities affecting Indigenous populations in Latin America, identify research gaps, and inform policy recommendations.

**Methods:**

Following PRISMA-ScR guidelines, we systematically searched PubMed, Embase, and Scielo for studies with data collected between May 2014 and May 2024. Studies were included if they examined health disparities among Indigenous populations in Latin America, contained a comparator related to disparities, and presented quantitative data. We excluded studies on oral health, risk factors, genetic disparities, health system access, and ecological studies, as well as non-research articles such as commentaries and letters to the editor. Data were synthesized narratively, summarizing key themes.

**Results:**

Of 1,116 identified articles, 35 met inclusion criteria, spanning nine Latin American countries. Most studies were cross-sectional (*n* = 18) or cohort-based (*n* = 16). Infectious diseases and malnutrition were the most studied topics, consistently showing higher incidence and mortality rates among Indigenous populations. Many papers highlighted COVID-19 disparities, with Indigenous groups experiencing higher incidence and mortality. Malnutrition, particularly stunting and anemia, was significantly more prevalent among Indigenous children and women. Studies on overweight and obesity showed mixed results. Chronic diseases, including chronic kidney disease and cardiovascular issues, showed notable disparities, while mental health and cancer were underrepresented.

**Conclusion:**

Indigenous populations in Latin America face a dual burden of infectious and chronic diseases, compounded by structural barriers such as poverty, geographic isolation, and systemic discrimination. Addressing these disparities requires culturally tailored interventions, structural reforms, and policy prioritization. This scoping review is limited by database restrictions, search term variability, language and time frame constraints, the absence of a methodological quality assessment, inconsistencies in defining Indigenous status, exclusion of grey literature, and a focus on disease prevalence rather than disparities in risk factors, diagnosis, and treatment, which may result in an incomplete representation of Indigenous health disparities in Latin America. Future research should incorporate mental health outcomes to provide a more comprehensive understanding of Indigenous health disparities.

**Supplementary Information:**

The online version contains supplementary material available at 10.1186/s12939-025-02495-2.

## Introduction

Health disparities, or inequities, are preventable differences in health outcomes that disproportionately affect disadvantaged populations compared with a reference group or population. These disparities impose substantial social and economic costs on individuals and society as a whole [1, 2]. They are shaped by various social and structural factors, including education, employment, geographic context, income level, gender, race, ethnicity, and poverty [1, 8]. Additionally, experiences of racism and discrimination contribute to inequities in healthcare access and quality, further undermining the well-being of affected individuals [1]. 

Latin America is one of the most diverse regions in the world, both ethnically and socioeconomically [3]. The region is characterized by substantial socioeconomic inequalities between and within countries, affecting healthcare delivery, access to health services, and population health outcomes [3, 4]. The region is also home to a significant portion of the world’s Indigenous population, with an estimated 33–40 million Indigenous people residing in Latin America and the Caribbean [5–7]. 

Indigenous groups in the region are incredibly diverse and are characterized by their cultures, languages, traditions, identities, and beliefs. Despite their historical importance, they have long been excluded, facing systemic discrimination that further exacerbates the social, economic, and health inequalities they face [5, 8]. Indigenous people constitute 40% of the rural population in Central and South America [9]. These populations represent some of the most disadvantaged groups in the region; approximately 43% of the Indigenous population in Latin America lives in poverty, more than twice the population of non-Indigenous backgrounds, and 25% live in extreme poverty [8]. In addition, Indigenous populations face significant healthcare inequities, including barriers to access and systemic obstacles within the healthcare system. These disparities are often exacerbated by geographical isolation, language barriers that hinder effective communication with healthcare professionals, and concerns about discrimination and prejudice. This marginalization has resulted in Indigenous populations falling behind their non-Indigenous counterparts across nearly all social determinants of health [8, 10–13]. 

Indigenous health disparities are a pressing public health issue that requires immediate attention due to the underrepresented state in which they remain, considering that their health remains at the greatest risk. Given the profound impact of inequitable social systems on Indigenous populations in Latin America, understanding what types of health disparities and conditions they cause on Indigenous populations is critical to developing systems where universal health coverage is achieved [11]. This scoping review aims to comprehensively map existing health disparities affecting Indigenous populations in Latin America, identify the areas of research that have been covered, and highlight gaps in the current literature.

Methods.

### Data sources and searches

The primary research question of this scoping review was ‘What are the health disparities experienced by Indigenous populations in Latin America?’ This scoping review was conducted following the PRISMA-ScR (Preferred Reporting Items for Systematic Reviews and Meta-Analyses extension for Scoping Reviews) guidelines, which provide a widely accepted methodological framework to enhance the transparency, reproducibility, and rigor of scoping reviews [14].

A comprehensive literature search was conducted in PubMed, Embase, and Scielo between June and July 2024. Specific search strategies were tailored for each database, including all of Latin America’s countries, “health disparities” or “inequities,” and other analogous terms. To search for Indigenous populations, our search strategy included the following terms: “Indigenous populations,” “Native population,” “aboriginal population,” “tribal population,” “pueblos originarios,” “Amerindian,” or “Ethnic group” and their Spanish equivalents (see search strategies in the supplemental material). The search was restricted by date, and the search strategies were conducted in Spanish and English. During this initial search, studies were not filtered by language or study type.

### Data Availability

The articles and documents supporting the conclusions of this review are available in the reference list of this article.

### Inclusion and exclusion criteria

We used the screening platform Covidence to manage citations and apply our eligibility criteria. For inclusion, we considered studies published between May 2014 and May 2024 in English, Spanish, or Portuguese that examined health disparities among Indigenous populations in any Latin American country. Studies needed to include a comparator related to health disparities and present quantitative data. For exclusion, we did not include studies on oral health, risk factors, genetic disparities, or health system access, as these were beyond the scope of our review, which focused on broader population-level health disparities. Additionally, we excluded ecological studies, as they did not allow for direct individual-level comparisons, and non-research articles, such as commentaries, correspondences, and letters to the editor, as they did not contain original data.

### Study selection

After the eligibility criteria were developed, we began the selection process. Covidence was then removed from any duplicate articles, and we completed level one screening, which included screening titles and abstracts on the basis of the inclusion criteria stated above. The screenings completed for this scoping review were performed in a masked, duplicate manner, in which reviewers resolved discrepancies through conflict resolution. When screening for titles and abstracts, we could vote on three options: to include, not include, or potentially include each study.

Once level one screening and conflict resolution were completed, we independently completed full-text reviews of the included abstracts. The characteristics of these studies were in accordance with the eligibility criteria. Nevertheless, we refined it so that ecological studies, geospatial studies, and genetic studies were excluded when performing the full-text reviews. Full-text reviews were successful by individually excluding studies based on an automated checklist provided by Covidence, which provides the following exclusion criteria: paper not available, wrong study design, wrong setting, wrong route of administration, wrong patient population, wrong outcomes, wrong intervention, wrong indication, wrong dose, wrong comparator, pediatric population, and adult population. After examining each article following this checklist, we identified any discrepancies in our choices for inclusion or exclusion decisions and for the specific exclusion criteria we selected.

### Data extraction

After the final round of full-text screening, we created a data extraction template for Covidence to capture critical information from the selected studies. The template included 12 study characteristics. These included study information, study design, country, setting, number of subjects, health outcomes, population, definition of ethnic identification, comparator, main findings, direction of inequity, and effect measures. We categorized ethnic identification as “not specified” for studies that did not define Indigenous status. Data extraction was performed in Covidence and then exported to an Excel site; data extraction was deemed complete once each reviewer reviewed the other extraction template.

### Findings

Our literature search identified a total of 1,116 studies from databases and registers, with the majority from Embase (*n* = 551) and PubMed (*n* = 520). After 70 duplicates were removed, we screened 1,046 studies by title and abstract and excluded 926 studies on the basis of predetermined criteria. This left 120 studies for full-text screening, 85 of which were further excluded for reasons such as wrong setting, outcomes, or population, among others. Ultimately, 35 studies were included in our data extraction and analysis review. The complete study selection process is illustrated in the PRISMA flow diagram (Table 1, 2).

**Table 1:**
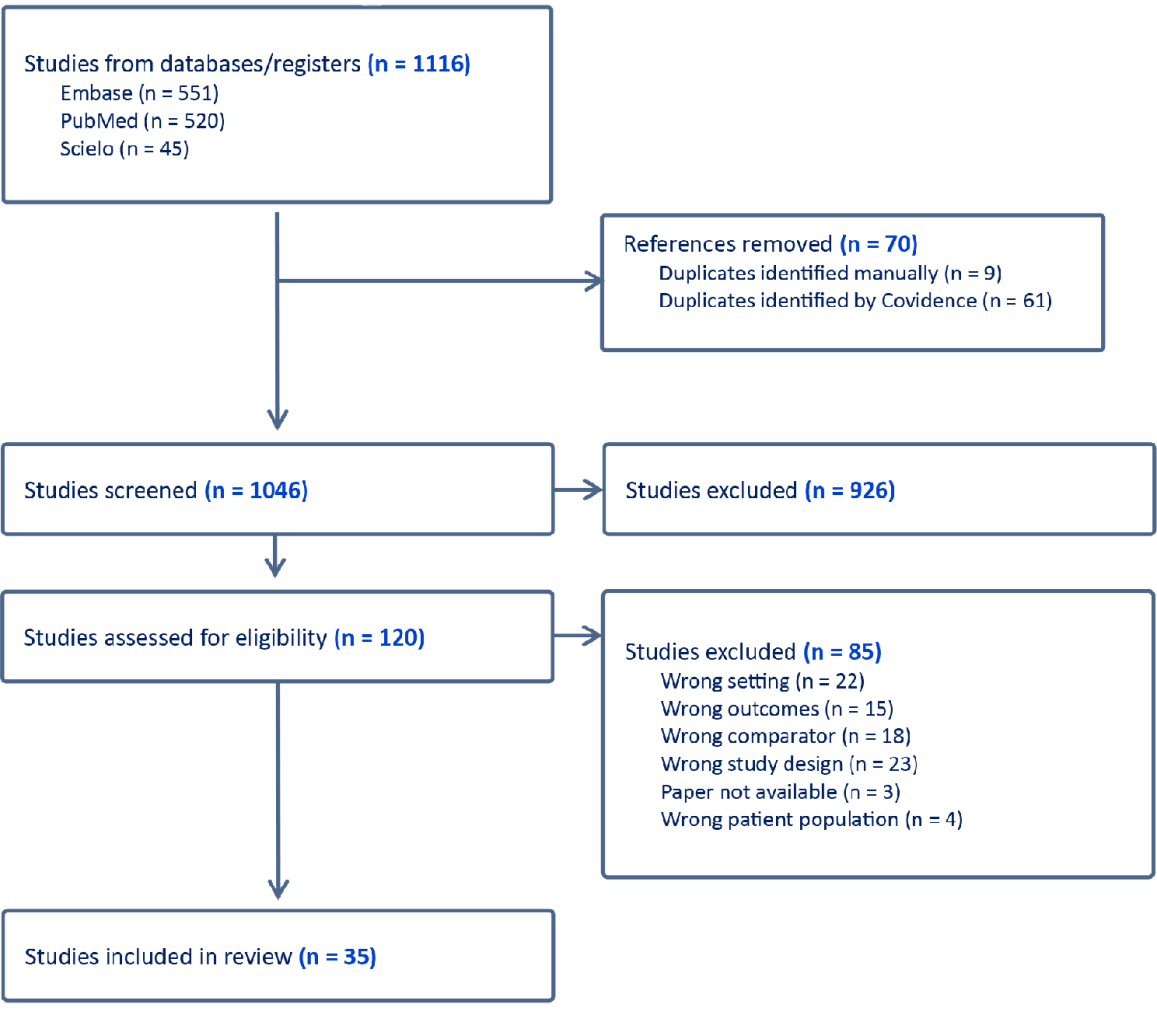
Search process showcased in a PRISMA flowchart for the scoping review

**Table 2:** Summary of included articles

Study ID	Study Design	Country	Years	Number of Subjects	Disease or health outcome	Definition of Ethnic ID	Main findings
Alarcón 2018	Cross-Sectional Study	Chile	2016–2017	558	HIV/AIDS	Surname-based and self-identification	Mapuche patients were generally younger, more likely to be heterosexual, and had lower educational and income levels compared to non-Mapuche patients. Additionally, they had lower median CD4(+) lymphocyte counts, indicating more advanced disease at the time of diagnosis. Specifically, Mapuche patients had a median CD4(+) count of 226 cells/mm³ (CI: 147.1–281.6), compared to 233 cells/mm³ (CI: 203–274.8) in non-Mapuche patients.
Alvear-Vega 2022	Cross-Sectional Study	Chile	2017	1,270,485	Malnutrition	Self-identification	The study observed that Indigenous children had a higher likelihood of experiencing both undernutrition and overnutrition. The relative risk ratio (RRR) for overnutrition was 1.17 (95% CI: 1.15–1.19), while the RRR for undernutrition was 1.09 (95% CI: 1.07–1.13).
Argoty-Pantoja 2021	Cohort Study	Mexico	2020	424,637	COVID-19	Language-based	The crude COVID-19 fatality rate was 64.8% higher in the Indigenous population compared to the non-Indigenous population, with a fatality rate of 29.97 per 1000 person-weeks for Indigenous individuals versus 18.18 for non-Indigenous individuals. Indigenous outpatients faced a significantly higher risk of death (HR = 1.63, 95% CI: 1.34–1.98) compared to non-Indigenous outpatients. The highest disparities were observed in the South Pacific region (HR = 2.35, 95% CI: 1.49–3.69) and in a subgroup of 13 states (HR = 1.66, 95% CI: 1.33–2.07).
Balda 2020	Cohort Study	Ecuador	2012–2016	6,334	Heart failure	Not specified	This study identified significant predictors of decreased heart failure (HF) mortality, including belonging to Native American or mixed-race populations. Native American ethnicity was associated with a lower mortality rate (β = 0.45, *p* < 0.01), while individuals of mixed race also exhibited a reduced mortality rate (β = 0.15, *p* = 0.01). These findings suggest that racial and ethnic factors may influence HF mortality rates.
Batis 2020	Cross-Sectional Study	Argentina, Bolivia, Brazil, Chile, Colombia, Ecuador, Guatemala, Mexico, Peru, Uruguay).	2005–2018	73,620	Malnutrition: overweight, obesity, stunting, and anemia (when available)	Self-identifications for Bolivia, Brazil, Ecuador, Colombia, Chile, Guatemala and language-based for Mexico and Peru.	Indigenous populations consistently exhibited higher prevalence of stunting/short stature and anemia across all age groups compared to non-indigenous populations. On average, indigenous populations had 19% points higher stunting/short stature and 6.7% points higher anemia prevalence than non-indigenous groups. The relationship between ethnicity and overweight/obesity was inconsistent. In some countries, indigenous populations had a higher prevalence, while in others, non-indigenous populations had higher rates
Cabrera 2022	Cohort Study	Chile	2012–2019	912	Chronic lymphocytic leuke- mia (CLL)	Surname-based	The incidence of Chronic Lymphocytic Leukemia (CLL) in the general Chilean population was 1.17 per 100,000 person-years, significantly higher than the 0.09 per 100,000 person-years observed in the Chilean Amerindian population. The 5-year overall survival (OS) rate for Chilean Amerindian patients was 29% (95% CI: 1–69%), compared to 57% (95% CI: 52–61%) in non-Amerindian Chilean patients, though this difference was not statistically significant (*p* = 0.28).
Cardoso 2023	Cohort Study	Brazil	2020–2022	2,459,844	COVID-19	Self-identification	Indigenous patients had significantly higher hospital mortality rates compared to White patients. In 2020, the odds of death for Indigenous patients were 1.99 (95% CI: 1.59–2.48) compared to White patients. Those using the public health system had an OR of 1.68 (95% CI: 1.42–1.97), while ICU admission increased the risk by 47% (OR = 1.47; 95% CI: 1.08–2.02). Additional risks included tomography (OR = 1.88; 95% CI: 1.43–2.46) and ventilatory support (OR = 1.80; 95% CI: 1.49–2.17).
Conde 2018	Cross-Sectional Study	Brazil	2015	16,556	Overweight, obesity, underweight	Self-identification	The study highlighted an increase in overweight and obesity prevalence among Black and Indigenous adolescents compared to previous years. While White adolescents had a higher overall prevalence of overweight, Indigenous adolescents demonstrated greater odds of being overweight, with a prevalence of 22.5% (OR = 1.02, 95% CI: 1.01–1.03). Additionally, individuals of Indigenous background exhibited the second-highest odds of underweight at 3.0% (OR = 1.00, 95% CI: 0.98–1.02), surpassed only by adolescents of Yellow skin color.
Contreras-Haro 2024	Cross-Sectional Study	Mexico	2023	378	Chronic kidney disease (CKD)	Self-identification and surname-based	The prevalence of chronic kidney disease (CKD) was significantly higher in the Wixárika group, with 15% of individuals affected compared to 4% in the mestizo group (*p* < 0.0001). Members of the Wixárika community exhibited lower levels of education and a higher frequency of alcoholism and elevated blood pressure compared to the mestizo population. Significant predictors of CKD included belonging to the Wixárika ethnic group, with an odds ratio (OR) of 14.27 (95% CI: 3.69–55.1; *p* < 0.0001), as well as older age (OR = 1.08; 95% CI: 1.03–1.13) and hypertension (OR = 9.93; 95% CI: 2.45–40.0).
Cuéllar 2022	Cohort Study	Ecuador	2020	87,762	Excess deaths of COVID-19	Self-identification	Indigenous populations had the highest excess death factor (EDF) of 2.2 (220% of expected deaths), compared to 1.36 (136%) in the Mestizo population. While death factors by sex and age in Indigenous groups were similar to the general population, females aged 20–50 had higher death factors than males. Unreported ethnic data post-July 2020 suggests Indigenous groups were disproportionately affected by COVID-19 beyond confirmed death counts.
Cuevas-Nasu 2019	Cross-Sectional Study	Mexico	2012 and 2018	2012: 7,141 2018: 2,439	Undernutrition: Stunting (chronic malnutrition), underweight, and wasting	Language-based	The logistic regression analysis indicated that children under five years of age who speak an Indigenous language had a 2.3-fold greater likelihood of being chronically undernourished (OR = 2.3; 95% CI: 1.3–4.0). Despite this, Indigenous children demonstrated a higher rate of dietary diversity, with 79.5% consuming a varied diet compared to only 20.5% of non-Indigenous children. This dietary diversity serves as a protective factor against chronic undernutrition. The prevalence of chronic undernutrition among Indigenous children under five in 2018 was 24.5% (95% CI: 17.8–32.6), highlighting persistent health disparities.
Curi-Quinto 2020	Cross-Sectional Study	Peru	2015	Children under 5 years: 22,833 Women of reproductive age (WRA): 33,503 (5,008 adolescents and 28,495 adults)	Malnutrition: overweight/obesity, wasting/underweight, stunting/short stature, and anemia.	Language-based	Indigenous children had higher prevalences of stunting (37.4%) and anemia (45.8%) compared to non-indigenous children (stunting 13.0%, anemia 30.9%), lower prevalence of overweight (3.7%) compared to non-indigenous children (9.2%). Indigenous adolescents had a higher prevalence of stunting (37.1%) compared to non-indigenous adolescents (21.7%). Indigenous adolescents had a lower prevalence of overweight/obesity (22.8%) compared to non-indigenous adolescents (31.7%), a higher prevalence of anemia (21.2%) compared to non-indigenous adolescents (18.7%). Indigenous adult women had a higher prevalence of short stature (50.4%) compared to non-indigenous women (33.4%), a lower prevalence of overweight/obesity (55.3%) compared to non-indigenous women (65.7%) and a higher prevalence of anemia (22.7%) compared to non-indigenous women (20.1%).
de Campos Gomes 2020	Cohort Study	Brazil	1996–2016	10,028	Down syndrome (DS)	Unspecified	Indigenous individuals, particularly women, showed higher mortality rates and lower survival rates for Down syndrome (DS), especially in the North and Midwest regions of Brazil. Indigenous status was strongly associated with increased mortality risk, more evident in women than men. Indigenous individuals with DS also tend to die at a younger age compared to other ethnic groups. Odds ratios varied significantly across regions, ranging from 8.829 to 0.07.
Góes 2024	Cohort Study	Brazil	2004–2015	20,665,005	Breast and cervical cancer	Self-identification	Mortality rates were highest among Indigenous women for cervical cancer (adjusted mortality rate ratio (MRR) = 1.80, 95% CI: 1.39–2.33) compared to White women. Low socioeconomic status (SES) magnified racial inequalities, with larger disparities among women with poorer household conditions and lower education levels. For breast cancer, Black women had the highest mortality rates (MRR = 1.10, 95% CI: 1.04–1.17), while Indigenous women had lower risks compared to White women (MRR = 0.63, 95% CI: 0.44–0.91).
Gopie 2021	Cohort Study	Suriname	2011–2015	662	Tuberculosis	Self-identification	The study revealed that the highest five-year tuberculosis (TB) incidence rates were observed among Indigenous populations (280 per 100,000; 95% CI: 187–374) and Creole populations (271 per 100,000) in Suriname. HIV coinfection was a significant risk factor for TB among Creole patients, with 38.2% being HIV positive. Indigenous TB patients were significantly younger than Creole patients. Even after adjusting for HIV status, Indigenous and Creole populations had higher TB incidence rates compared to other ethnic groups. The elevated TB rates among Indigenous individuals were attributed to factors like poverty and limited access to healthcare, particularly in remote areas.
Hallal 2020	Cross-Sectional Study	Brazil	2020	First survey: 25,025 Second survey: 31,165	COVID-19	Self-identification	The seroprevalence of SARS-CoV-2 in Brazil rose from 1.6% in May to 2.8% in June 2020, with significant regional disparities, notably higher levels along the Amazon River in the northern region. Indigenous people demonstrated a markedly higher seroprevalence (6.3%) compared to White individuals (1.4%), with an odds ratio (OR) of 1.87 (95% CI: 1.18–2.96). Higher seroprevalence was associated with lower socioeconomic status, crowded living conditions, and being in the 20–59 age group. Furthermore, individuals in the poorest quintile had more than double the seroprevalence of those in the wealthiest quintile in both surveys.
Horta 2021	Cross-Sectional Study	Brazil	2020	89,397	COVID-19	Self-identification	The study highlighted a significantly higher prevalence of SARS-CoV-2 antibodies among Indigenous individuals, with a prevalence ratio of 4.71 (95% CI: 3.65–6.08) compared to White individuals. Similarly, Black and Brown individuals showed higher antibody prevalence rates compared to Whites. There was a notable inverse relationship between wealth and antibody prevalence, as poorer individuals were more likely to have antibodies. Education levels also impacted antibody prevalence, with individuals possessing 12 or more years of schooling exhibiting lower prevalence rates.
Ibarra-Nava 2021	Cross-Sectional Study	Mexico	2020	416,546	COVID-19	Language-based	The study reveals a higher mortality rate and hospitalization burden among Indigenous populations compared to non-Indigenous groups in both public and private healthcare sectors. Most Indigenous individuals are uninsured or depend on public health systems. Municipalities with higher Indigenous populations had significantly fewer healthcare resources, with 63 clinics, 31 beds, and 86 doctors per 100,000 individuals, compared to municipalities with fewer Indigenous residents, which had 377 clinics, 336 beds, and 670 doctors per 100,000. Indigenous peoples faced an overall COVID-19 mortality rate of 16.5%, compared to 11.1% for non-Indigenous groups. Among hospitalized patients, Indigenous mortality was 37.1%, slightly higher than the 36.3% observed in non-Indigenous patients. Mortality was significantly elevated for Indigenous individuals receiving only ambulatory care (OR 1.55, 95% CI: 1.24–1.92). Additionally, Indigenous individuals were more likely to die outside hospital settings (3.7%) compared to their non-Indigenous counterparts (1.7%). The overall odds of COVID-19 mortality for Indigenous peoples were also higher (OR 1.13, 95% CI: 1.03–1.24).
Kain 2019	Cohort Study	Chile	2011–2017	483,509	Overweight and obesity	Self-identification	The study found that Indigenous children in Chile are at a significantly higher risk of developing overweight or obesity by the time they enter first grade. Approximately 30% of normal-weight preschoolers transitioned to overweight or obesity during this period. Key factors contributing to this trend include being of Indigenous ethnicity, with odds ratios of 1.18 (95% CI: 1.11–1.23) for boys and 1.08 (95% CI: 1.02–1.13) for girls. Additional predictors include attending highly vulnerable schools (OR 1.06 for boys and OR 1.05 for girls), having a mother with only primary education (OR 1.07 for boys and OR 1.19 for girls), and being born with a high birth weight (OR 1.46 for boys and OR 1.47 for girls).
Lapo-Talledo 2024 A	Cohort Study	Ecuador	2015–2022	1,118,842	Maternal mortality	Not specified	Maternal mortality peaked in 2020 at 32.22 deaths/100,000 live births before declining to 18.94 in 2022. Ethnic minorities in the “Other” category had significantly higher delivery-related mortality (AOR = 9.59, 95% CI: 6.98–13.18), while Indigenous women showed no significant difference compared to Mestizo women (AOR = 0.61, 95% CI: 0.19–1.93). Higher mortality was also linked to private healthcare (AOR = 1.99) and emergency caesareans (AOR = 7.49).
Lapo-Talledo 2024 B	Cohort Study	Ecuador	2015–2022	31,616	Dengue	Self-identification	Indigenous people demonstrated a significantly lower risk of complicated dengue hospitalization (aRRR = 0.44, 95% CI: 0.34–0.55) and in-hospital death (OR = 0.72, 95% CI: 0.10–5.25) compared to Mestizos.
Little 2023	Cohort Study	Mexico	2020–2022	10,487,563	COVID-19	Self-identification and language-based	Indigenous individuals faced a nearly two-fold unadjusted risk of COVID-19 fatality compared to non-Indigenous individuals (OR = 1.92, 95% CI: 1.86–1.99). However, after full adjustment, the risk was 4% higher (OR = 1.04). Marginalized Indigenous individuals were 1.29 times less likely to be admitted to the ICU and 1.56 times less likely to receive mechanical ventilation. Marginalization increased COVID-19-related death probability by 1.51-fold. Pre-existing conditions, including diabetes, pneumonia, hypertension, and obesity, further amplified COVID-19 mortality risks for Indigenous individuals.
Mamani Ortiz 2019	Cross-Sectional Study	Bolivia	2015 and 2016	5,758	Abdominal obesity	Self-identification	The study found that Indigenous individuals had lower obesity prevalence compared to Mestizo individuals, but experienced less favorable socioeconomic conditions, such as a higher proportion of people with no formal education (9.98%). The disparity in obesity prevalence between Mestizo men and Indigenous women (7.26% higher for Mestizo men) was attributed to ethnic differences. Behavioral risk factors, particularly alcohol consumption and smoking, were more prevalent among Mestizos, while Indigenous individuals displayed healthier eating habits. Abdominal obesity prevalence was highest among Mestizo men (35.01%), followed by Mestizo women (30.71%), Indigenous women (27.75%), and Indigenous men (25.38%). Gender disparities were observed, with Mestizo men having a 4.3% higher prevalence compared to Mestizo women, and ethnic disparities showed a 9.18% higher obesity prevalence in Mestizo men compared to Indigenous men.
Mazariegos 2020	Cross-Sectional Study	Guatemala	2014–2015	Children younger than 5 years: *n* = 11,962. Adolescent girls aged 15–19 years: *n* = 1,086. Women of reproductive age aged 20–49 years: *n* = 11,354.	Malnutrition	Self-identification	The findings reveal notable disparities in nutritional status across different age groups and socioeconomic strata. Among children under 5 years of age, stunting was significantly more prevalent in Indigenous children, who were 1.7 times more likely to experience stunting compared to their non-Indigenous peers. Conversely, overweight and obesity were more common among wealthier, non-Indigenous children, with non-Indigenous children having a 1.3 times higher prevalence of overweight or obesity (*p* < 0.05). For adolescent girls, similar patterns emerged, with Indigenous girls showing a 1.7 times higher prevalence of stunting compared to non-Indigenous girls. In contrast, non-Indigenous girls were 1.7 times more likely to exhibit overweight or obesity compared to Indigenous girls (*p* < 0.05). Among women of reproductive age, Indigenous women had a 1.6 times higher prevalence of stunting compared to non-Indigenous women. However, overweight and obesity were 1.3 times more prevalent among non-Indigenous women (*p* < 0.05).
Muñoz-Del-Carpio-Toia 2024	Cohort Study	Peru	2016–2021	85,905	Childhood anemia	Self-identification	Quechua, Aymara, Amazonian natives, and other Indigenous children had a higher prevalence of childhood anemia compared to Mestizo children, both before and during the pandemic. Aymara children showed the highest prevalence (PR = 1.35, 95% CI: 1.27–1.44, *p* < 0.001), followed by other Indigenous children (PR = 1.29, 95% CI: 1.05–1.57, *p* = 0.013), Amazonian natives (PR = 1.20, 95% CI: 1.12–1.28, *p* < 0.001), and Quechua children (PR = 1.11, 95% CI: 1.07–1.15, *p* < 0.001). White children demonstrated a lower prevalence of anemia compared to Mestizo children.
Mujica-Coopman 2020	Cross-Sectional Study	Chile	2016–2017	5,082	Malnutrition	Self-identification	The study found that 12.4% of low SES adults identified as Indigenous, compared to 3% of high SES adults. Obesity was more frequent among Indigenous young women (55.8%) compared to non-Indigenous women (37.2%), while there was no significant difference among older women. Excess weight in men did not vary significantly by SES or ethnicity. However, short stature was more prevalent among Indigenous men (21.5%) compared to non-Indigenous men (8.2%).
Oliveira 2021	Cohort Study	Brazil	2020–2021	82,055	COVID-19	Self-identification	Indigenous children and adolescents had a significantly higher risk of death compared to White children (HR: 3.36; 95% CI: 2.15–5.24). Mortality rates were also higher in the Northeast and North regions of Brazil compared to the Southeast region. Additionally, the presence of pre-existing medical conditions increased the risk of death, with hazard ratios of 2.96 for individuals with one condition and up to 7.28 for those with three or more conditions.
Pereira 2022	Cohort Study	Brazil	2020–2021	Mild/moderate cases: 70,056,602 Severe cases: 2,801,380	COVID-19	Self-identification	Indigenous individuals had the highest odds of death (OR = 1.42, 95% CI 1.31–1.54) compared to White individuals. Men had higher odds of death than women (OR = 1.14, 95% CI 1.13–1.15), and residents of deprived municipalities also had increased odds (OR = 1.38, 95% CI 1.36–1.40). The risk of death was particularly high for patients requiring ICU admission (OR = 5.19, 95% CI 5.14–5.24).
Ponce-Alcala 2021	Cross-Sectional Study	Mexico	2016	5,456	Obesity	Self-identification and language-based	The prevalence of obesity was significantly higher among women (38.7%) compared to men (28.6%). Severe household food insecurity was strongly associated with an increased likelihood of obesity in women, with an odds ratio (OR) of 2.36 (*P* = 0.001). Similarly, abdominal obesity was more prevalent in women (87.2%) than men (64.1%), with a significant correlation between severe food insecurity and abdominal obesity among women. Indigenous adults, particularly women, experienced higher levels of food insecurity and greater proportions of obesity compared to non-Indigenous groups. The proportion of household food security among Indigenous individuals was only 12.3% (95% CI: 7.9%, 18.8%; *n* = 594), much lower than the 30.4% (95% CI: 27.9%, 33.0%; *n* = 4,862) reported for non-Indigenous individuals.
Rebouças 2022	Cohort Study	Brazil	2012–2018	19,515,843	All-cause child mortality and cause-specific mortality (diarrhea, malnutrition, pneumonia, accidents, and ill-defined causes).	Self-identification	Indigenous children experienced the highest risk of death before the age of 5 years compared to other ethnic groups, with an adjusted hazard ratio (HR) of 1.98 (95% CI: 1.92–2.06) compared to White children. Post-neonatal mortality was significantly higher among Indigenous children (HR: 3.88; 95% CI: 3.68–4.10). The highest mortality risks were for malnutrition (HR: 16.39; 95% CI: 12.88–20.85), diarrhea (HR: 14.28; 95% CI: 12.25–16.65), and pneumonia (HR: 6.49; 95% CI: 5.78–7.27). Indigenous mothers had the lowest proportion of prenatal consultations (28.6% attended less than three), further emphasizing disparities in healthcare access and outcomes.
Ronquillo De Jesús 2022	Cross-Sectional Study	Mexico	2020	1,037,567	COVID-19	Not specified	The case fatality rate for COVID-19 was notably higher among the Indigenous population (14.7%) compared to the non-Indigenous population (9.7%). Severe cases constituted 16.6% of COVID-19 cases in the Indigenous group, whereas they made up only 11.0% in the non-Indigenous group. The prevalence of comorbidities, such as hypertension, obesity, or diabetes, was also higher among Indigenous individuals (42.7%) compared to non-Indigenous individuals (35.3%), although non-Indigenous individuals had a higher prevalence of tobacco use. More than 80% of fatalities occurred in individuals aged 50 or older across both groups. The cumulative incidence rate for the Indigenous population was 94.4 per 100,000, with a death rate of 13.9 per 100,000, while for the non-Indigenous population, the cumulative incidence rate was 900.3 per 100,000, with a death rate of 87.1 per 100,000.
Salas-Ortiz 2024	Cross-Sectional Study	Mexico	2020–2021	4,829,071	COVID-19	Language-based	The study highlights the inequities faced by Indigenous populations in medical infrastructure, marginalization, and healthcare outcomes during the COVID-19 pandemic. Indigenous individuals experienced higher rates of hospitalization, early death within 5 days, and overall mortality compared to non-Indigenous populations. Equalizing the distribution of comorbidities between Indigenous and non-Indigenous groups could reduce disparities in hospitalizations by 40.6%, early deaths by 42%, and total deaths by 48.4%. Indigenous communities had poorer access to healthcare infrastructure, with an average of 3.45 medical offices and 9.34 hospital beds per community, compared to 5.31 medical offices and 15.52 hospital beds in non-Indigenous areas. Municipal marginalization was slightly lower for Indigenous communities (0.86) than non-Indigenous ones (0.93). Urban localities were less common in Indigenous areas (15.15% compared to 28.11%). Regarding COVID-19 outcomes, 24.9% of Indigenous individuals were hospitalized compared to 12.9% of non-Indigenous individuals. Mortality within 5 days was 5% for Indigenous individuals, more than double the 2.3% rate among non-Indigenous populations. Total mortality was 9.8% for Indigenous individuals, compared to 5.1% for non-Indigenous populations.
Silva 2017	Cross-Sectional Study	Brazil	2015	4000	Depression	Self-identification	Indigenous participants in the study were the least represented but showed the highest prevalence of depressive symptoms at 17.1%. They were three times more likely to exhibit depressive symptoms compared to White participants. Most Indigenous individuals with depressive symptoms were women, belonged to a low social class, and were engaged in informal work or were unemployed. The prevalence ratio for depressive symptoms among Indigenous participants compared to White participants was 2.56 (95% CI: 1.24–5.30).
Soto 2019	Case-Control Study	Chile	2017–2018	104	Incident stroke	Self-identification	This study found no association between Mapuche ethnicity and stroke. Although control variables such as hypertension, overweight/obesity, low socioeconomic status, rurality, diabetes, and smoking were associated with either stroke (outcome variable) or Mapuche ethnicity (exposure variable), none altered the effect of ethnicity on stroke. The odds ratio (OR) for Mapuche ethnicity and stroke was 0.75 (95% CI: 0.35–1.62, *p* = 0.47), indicating no significant association.
Vinueza Veloz 2023	Cross-Sectional Study	Ecuador	2018	89,212	Malnutrition	Self-identification	The study found variations in BMI across different demographics and ethnic groups. Women had a 1.03 kg/m² higher BMI than men, and BMI increased by 0.04 kg/m² per year of age. Married individuals had a 1.14 kg/m² higher BMI compared to single individuals. Indigenous participants had a BMI 0.78 kg/m² lower than White participants, while Montubio and Afro-American individuals had 0.37 and 0.61 kg/m² higher BMI, respectively. Urban residents also showed a 0.41 kg/m² higher BMI than rural residents. The multivariate analysis indicated a coefficient of -0.79 (95% CI: -1.02, -0.56) for Indigenous nutritional status, reflecting lower BMI levels.

### Included studies

The included studies spanned various countries across Latin America, with research conducted in Ecuador [5], Brazil [10], Mexico [8], Chile [6], Peru [2], Suriname [1], Bolivia [1], Guatemala [1], and a multicounty study involving several Latin American nations. Table 1 summarizes characteristics for included studies.

Study designs varied, with the majority utilizing cross-sectional (*n* = 18), cohort (*n* = 16), and case‒control (*n* = 1) studies. Most studies (57%) have used national datasets, whereas others have relied on regional or local data sources. The highest concentration of research was between 2019 and 2021, with five studies published in 2019, 8 studies in 2020, and 6 in 2021.

The studies included a wide range of health areas, with infectious diseases and malnutrition being the most studied topics. In the case of infectious diseases, COVID-19 was the most common focus. All the included studies revealed that Indigenous populations consistently had higher COVID-19 mortality rates and prevalence rates than non-Indigenous populations did. In Brazil, Indigenous individuals had a prevalence ratio (PR) of 4.71 (95% CI: 3.65–6.08) for COVID-19 infection, with higher odds and risk of COVID-19 mortality reported in five studies: OR 1.87 (95% CI: 1.18–2.96), OR 1.42 (95% CI: 1.31–1.54), OR 1.99 (95% CI: 1.59–2.48), and HR of 3.36 (95% CI: 2.15–5.24) [15–19]. A study performed in Ecuador reported that Indigenous populations had an excess death factor of 2.2, compared with 1.36 for the predominant ethnic group [20]. Five studies in Mexico reported adverse outcomes for Indigenous individuals, including a 5% higher fatality rate, a higher death rate (87.1 per 100,000 vs. 13.9 per 100,000 for non-Indigenous individuals), higher odds of dying from COVID-19 (OR 1.92 [95% CI: 1.86–1.99] and OR 1.13 [95% CI: 1.03–1.24]) and a higher risk for COVID-19 fatality (HR 1.63 [95% CI: 1.34–1.98]) [21–23]. 

The incidence of tuberculosis was higher among Indigenous groups in Suriname, with an incidence rate of 280 per 100,000 people (95% CI: 187–374) [24]. In Chile, Indigenous people living with HIV had lower white cell counts at diagnosis than non-Indigenous individuals did (Mapuche: 226 cells/mm³ [95% CI: 147.1–281.6] vs. Non-Mapuche: 233 cells/mm³ [95% CI: 203–274.8]) [25]. Indigenous children in Brazil were found to have higher mortality rates from diarrhea and pneumonia than White children did, with HRs of 14.28 (95% CI: 12.25–16.65) and 6.49 (95% CI: 5.78–7.27), respectively [26]. One study from Ecuador reported that Indigenous people had lower odds of complicated dengue hospitalization than did Mestizo individuals did (OR 0.44 [95% CI: 0.34–0.55]) [27]. 

Thirteen studies included malnutrition, with most finding consistently higher rates of various undernutrition outcomes in Indigenous groups than in non-Indigenous groups and mixed findings for overnutrition. Many of the studies included more than one outcome. Indigenous children across multiple countries had a significantly higher prevalence of stunting and anemia. A multicountry study revealed that, on average, Indigenous populations had a 6–7% point higher incidence of anemia [28]. In Peru, anemia incidence was reported to be significantly greater among Quechua children (PR 1.11, 95% CI: 1.07–1.15), Aymara children (PR 1.35, 95% CI: 1.27–1.44), Amazonian native children (PR 1.20, 95% CI: 1.12–1.28), and other Indigenous children (PR = 1.29, 95% [CI: 1.05–1.57]) than among non-Indigenous children [29]. A study in Peru revealed that Indigenous children under 5 years of age, adolescent women, and adult women had a greater prevalence of anemia than their non-Indigenous counterparts did (PR = 1.19 [95% CI: 1.1–1.3]; PR 1.57 [95% CI: 1.1–2.2], and PR 1.28 [95% CI: 1.1–1.5], respectively) [30]. Indigenous adolescents in Brazil had a greater prevalence of underweight (3.0%, OR 1.00, 95% CI: 0.98–1.02), and in Ecuador, a study revealed that Indigenous populations had a negative coefficient for nutritional status (-0.79, 95% CI: -1.02, -0.56) [31, 32]. Indigenous children in Brazil had a significantly greater risk of mortality from malnutrition (HR 16.39, [95% CI: 12.88–20.85]) [26]. 

In terms of overweight and obesity, the results were mixed. For example, Indigenous children and adolescents in Brazil and Chile had higher rates of overweight/obesity (OR 1.02 [95% CI: 1.01–1.03] and RRR 1.17 [95% CI: 1.15–1.19], respectively) [31, 33]. However, in Guatemala, non-Indigenous children had a 1.3-fold greater prevalence of overweight/obesity than Indigenous children did (*p* < 0.05) [34]. 

Cardiovascular and chronic diseases were studied in Ecuador and Chile, with heart failure mortality rates being lower among Indigenous populations in Ecuador (β = 0.45, *p* < 0.01), whereas no difference was detected in stroke incidence in Chile (OR 0.75 [95% CI: 0.35–1.62]) [35, 36]. In Mexico, the prevalence of chronic kidney disease (CKD) was greater in the Wixárika (Huichol) Indigenous group (15%) than in the Mestizo population (4%) (OR = 14.27, *p* < 0.0001) [37]. 

Two studies included cancer outcomes. A study in Brazil revealed that compared with Indigenous women, white women had a higher mortality rate for cervical cancer (MRR = 1.80 [95% CI 1.39–2.33]) and a lower mortality rate ratio for breast cancer (MRR = 0.63 [95% CI 0.44–0.91]) [38]. A study in Indigenous groups in Chile revealed that they had a lower incidence of chronic lymphocytic leukemia (CLL) than other racial and ethnic groups did (0.09 per 100,000 person-years vs. 1.17 per 100,000 person-years) [39]. 

In Brazil, a mental health study revealed that Indigenous populations had a significantly greater prevalence of depressive symptoms than other ethnic groups did. Indigenous participants exhibited the highest proportion of depressive symptoms, with a prevalence of 17.1% [40]. Compared with White participants, Indigenous individuals were approximately 2.5 times more likely to have depression (PR 2.56 [95% CI: 1.24–5.30]) [40]. Maternal health was also explored in Ecuador, where no significant difference in maternal mortality was observed between Indigenous and non-Indigenous populations [41]. 

Discussion.

This scoping review revealed significant health disparities affecting Indigenous populations across nine countries in Latin America. Infectious diseases, particularly COVID-19 and malnutrition, are the areas of health that have been most studied. Compared with non-Indigenous populations, Indigenous populations consistently presented higher rates of COVID-19 incidence and mortality, with studies in Brazil, Mexico, and Ecuador reporting significantly worse outcomes. Similarly, malnutrition disproportionately affects Indigenous children and women, with higher rates of stunting and anemia observed across multiple studies. Chronic diseases have been less frequently studied, but notable disparities have been identified, including higher prevalence rates of chronic kidney disease and cardiovascular health issues among Indigenous groups. Mixed findings emerged in terms of overnutrition outcomes, with some studies reporting higher rates of overweight and obesity in Indigenous populations than in non-Indigenous populations.

Our findings suggest that health disparities among Indigenous populations in Latin America highlight the dual burdens of infectious and chronic diseases. While infectious diseases remain a leading cause of mortality and morbidity, the emergence of chronic diseases such as obesity and cardiovascular illnesses is becoming increasingly apparent. This double burden poses a significant challenge, as these communities navigate health systems that are ill equipped to address both communicable and noncommunicable diseases simultaneously. The trajectory of obesity, observed in wealthier countries as it transitioned from a disease of affluence to one of poverty, is evident in Indigenous populations of Latin America. In our review, studies in higher-income countries such as Brazil and Chile reported higher rates of obesity among Indigenous groups. In contrast, lower-income countries such as Guatemala presented lower rates of overnutrition but persistently high rates of undernutrition, such as stunting and anemia. Importantly, this pattern may also reflect gaps in research, as the focus has traditionally been on infectious diseases, potentially overlooking the growing impact and disparities of chronic diseases.

Our review also revealed significant disparities in COVID-19 outcomes among Indigenous populations in Latin America, mirroring patterns observed in high-income countries [42–44]. These findings emphasize the need to prioritize Indigenous populations in public health emergency planning and interventions. Structural factors such as poverty, geographic isolation, and systemic discrimination place Indigenous populations at increased risk for emerging infectious diseases. However, these vulnerabilities were compounded by systemic failures, as Indigenous groups were often overlooked in the allocation of health resources and interventions. Studies conducted in Mexico and Guatemala highlighted inequities in COVID-19 vaccination access and uptake, with Indigenous populations having lower odds of being vaccinated despite clear early evidence of their elevated risk for severe disease and mortality [45, 46]. These disparities underscore the need for equitable and culturally sensitive health policies to ensure that Indigenous communities are prioritized in public health responses to ongoing and emerging health crises.

Only one study on mental health was included in our review, underscoring a significant gap in research on mental health disparities among Indigenous populations in Latin America. During our screening process, we identified additional papers and reports highlighting the critical need for research in this area. However, these studies were excluded because they either predated our inclusion period or utilized study designs, such as ecological studies, that did not meet our criteria. For example, an ecological study revealed that suicide rates among Indigenous Brazilians in 2020 were more than two and a half times higher than those for the overall Brazilian population (17.57 versus 6.35 suicide deaths per 100,000 inhabitants, respectively) [47]. A systematic review conducted in 2017 on suicide among Indigenous populations in the region also identified suicide as a major concern [48, 49]. None of the studies included in that review utilized data published after 2014, making them ineligible for inclusion in our scoping review. The authors of the 2017 review also highlighted a lack of publications on Indigenous suicides from countries with the largest relative Indigenous populations, such as Bolivia, Guatemala, and Mexico, findings that mirror our own. Furthermore, a global systematic review on suicide among Indigenous peoples published in 2018 included only four studies from Latin America, all of which were conducted in Brazil [49]. These findings collectively emphasize the urgent need for more comprehensive and updated research on mental health disparities, particularly suicide, among Indigenous populations in the region.

Globally, these patterns are not unique to Latin America. Indigenous populations in high-income countries such as Canada, Australia, and New Zealand face similar systemic barriers to health equity, including exclusion from healthcare access, systemic racism, and historical marginalization [50–53]. Initiatives such as Australia’s “Closing the Gap” program aim to reduce disparities but have faced challenges due to inconsistent political commitment and underfunding [54, 55]. Similarly, fragmented healthcare systems in Canada often lead to delays and inequities for First Nations communities [56]. Resource constraints amplify these challenges in Latin America, underscoring the need for innovative, community-driven solutions.

Despite these challenges, initiatives such as Colombia’s Anas Wayúu health system demonstrate the potential of Indigenous-led healthcare models that integrate traditional medicine with modern practices, addressing geographic and linguistic barriers through culturally sensitive care [57–59]. These programs offer valuable lessons in bridging healthcare gaps, but their success relies on sustained funding, robust policy frameworks, and collaboration between governments, Indigenous communities, and international partners. Addressing health disparities among Indigenous populations requires structural reforms in healthcare systems and targeted interventions that respect and incorporate Indigenous knowledge and practices [60]. 

Despite its strengths, this scoping review has limitations. The systematic search strategy employed may not have captured all relevant studies due to database limitations, variability in search terms, and publication language restrictions. The absence of methodological quality assessment or bias evaluation limits the robustness of individual findings. The predefined time frame potentially excluded relevant older studies, such as those we experienced with mental health papers. Variations in how Indigenous status was defined across studies introduce inconsistencies, and the focus on disease prevalence excluded disparities in risk factors, diagnosis, and treatment. Another limitation is the exclusion of grey literature, such as thesis, government reports, and community-based research, which often contain valuable insights into Indigenous health disparities. Given that much research with Indigenous populations may not be published in indexed journals, this could have led to an incomplete representation of existing evidence. Future reviews may benefit from incorporating grey literature sources to provide a more comprehensive understanding of Indigenous health disparities in Latin America. Nonetheless, this review highlights critical gaps, emphasizing the need for future research to adopt a broader, more inclusive scope.

In conclusion, this review identifies health disparities experienced by Indigenous populations in Latin America, driven by systemic discrimination, socioeconomic deprivation, and structural barriers within healthcare systems. Ongoing and emerging infectious diseases and malnutrition remain significant concerns, while the growing prevalence of chronic diseases signals a troubling double burden. Addressing these disparities demands a comprehensive approach that combines targeted interventions with structural reforms to promote equity. Policymakers, researchers, and healthcare providers must collaborate to ensure that Indigenous populations are prioritized, fostering sustainable health equity and achieving universal health coverage.

## Electronic supplementary material

Below is the link to the electronic supplementary material.

Supplementary Material 1

## Data Availability

No datasets were generated or analysed during the current study.
