# Supplementary material for: Health disparities among indigenous populations in Latin America: a scoping review
Source: Int J Equity Health. 2025 Apr 30;24:119. doi: 10.1186/s12939-025-02495-2 (PMC12044809; doi:10.1186/s12939-025-02495-2)
Supplement: Supplementary file 1 — Supplementary Material 1 [file 12939_2025_2495_MOESM1_ESM.docx]

Search strategy:

Pubmed:

("Health Status Disparities"[MeSH] OR "Healthcare Disparities"[MeSH] OR "Health Equity"[MeSH] OR "Health Inequities"[tiab] OR "Health Inequality"[tiab] OR "Health Disparities"[tiab] OR "Social Determinants of Health"[MeSH] OR "Socioeconomic Factors"[MeSH] OR "Access to Health Care"[MeSH] OR "Healthcare Disparities"[tiab] OR "Social Inequity"[tiab]) AND ("Indigenous Peoples"[MeSH] OR "Indigenous"[tiab] OR "Native"[tiab] OR "Aboriginal"[tiab] OR "Pueblos Originarios"[tiab] OR "Ethnic Groups"[MeSH])

AND ("Latin America"[MeSH] OR "Central America"[MeSH] OR "South America"[MeSH] OR "Argentina"[MeSH] OR "Bolivia"[MeSH] OR "Brazil"[MeSH] OR "Chile"[MeSH] OR "Colombia"[MeSH] OR "Costa Rica"[MeSH] OR "Cuba"[MeSH] OR "Dominican Republic"[MeSH] OR "Ecuador"[MeSH] OR "El Salvador"[MeSH] OR "Guatemala"[MeSH] OR "Honduras"[MeSH] OR "Mexico"[MeSH] OR "Nicaragua"[MeSH] OR "Panama"[MeSH] OR "Paraguay"[MeSH] OR "Peru"[MeSH] OR "Uruguay"[MeSH] OR "Venezuela"[MeSH])

Scielo:

("disparidades en salud" OR "desigualdades en salud" OR "equidad en salud" OR "inequidades en salud" OR "diferencias en salud" OR "determinantes sociales de la salud" OR "factores socioeconómicos" OR "acceso a la atención médica" OR "inequidad social") AND ("pueblos indígenas" OR "indígenas" OR "nativos" OR "aborígenes" OR "pueblos originarios" OR "grupos étnicos") AND ("América Latina" OR "Sudamérica" OR "Centroamérica" OR "Caribe" OR "Argentina" OR "Bolivia" OR "Brasil" OR "Chile" OR "Colombia" OR "Costa Rica" OR "Cuba" OR "República Dominicana" OR "Ecuador" OR "El Salvador" OR "Guatemala" OR "Honduras" OR "México" OR "Nicaragua" OR "Panamá" OR "Paraguay" OR "Perú" OR "Uruguay" OR "Venezuela")

Embase:

('health care disparity'/exp OR 'health status disparity'/exp OR 'health equity'/exp OR 'health inequity'/exp OR 'health inequality'/exp OR 'health gap' OR 'health differences' OR 'socioeconomic factor'/exp OR 'social determinant of health' OR 'health care access'/exp OR 'social inequity'/exp) AND ('indigenous population'/exp OR 'native population' OR 'aboriginal population' OR 'tribal population'/exp OR 'pueblos originarios' OR 'amerindian' OR 'ethnic group'/exp) AND ('latin america'/exp OR 'south america'/exp OR 'central america'/exp OR 'caribbean'/exp OR 'argentina'/exp OR 'bolivia'/exp OR 'brazil'/exp OR 'chile'/exp OR 'colombia'/exp OR 'costa rica'/exp OR 'cuba'/exp OR 'dominican republic'/exp OR 'ecuador'/exp OR 'el salvador'/exp OR 'guatemala'/exp OR 'honduras'/exp OR 'mexico'/exp OR 'nicaragua'/exp OR 'panama'/exp OR 'paraguay'/exp OR 'peru'/exp OR 'uruguay'/exp OR 'venezuela'/exp) AND (2014:py OR 2015:py OR 2016:py OR 2017:py OR 2018:py OR 2019:py OR 2020:py OR 2021:py OR 2022:py OR 2023:py OR 2024:py) AND ([embase]/lim OR ([medline]/lim NOT ([embase classic]/lim AND [medline]/lim)))
